# Methylene Blue for the Treatment of Refractory Hypotension in Polysubstance Overdose

**DOI:** 10.7759/cureus.78238

**Published:** 2025-01-30

**Authors:** Maranda Jordan

**Affiliations:** 1 Emergency Medicine, Summa Health, Akron, USA

**Keywords:** calcium channel blocker overdose, calcium channel blocker toxicity, drug overdose, hypotension, hypotension treatment, medication overdose, methylene blue, methylene blue infusion, polysubstance use, refractory hypotension

## Abstract

Methylene blue is commonly used for methemoglobinemia and vasoplegia following coronary artery bypass procedures but has recently been investigated as a treatment for refractory hypotension. One such cause of refractory hypotension is overdose of calcium channel blocker medications. This case presents a patient with polysubstance overdose and refractory hypotension that improved with methylene blue infusion. This 67-year-old female presented to the ED following an intentional toxic ingestion of multiple substances, including metoprolol, amlodipine, mirtazapine, pantoprazole, quetiapine, levetiracetam, melatonin, and levothyroxine. The patient’s hypotension remained unresponsive to fluids, calcium chloride, calcium gluconate, and multiple pressure support medications, including norepinephrine, epinephrine, vasopressin, and phenylephrine. Insulin and dextrose, as well as methylene blue were initiated for severe refractory hypotension unresponsive to other measures. Methylene blue inhibits guanylate cyclase, which decreases the production of cyclic guanosine monophosphate. This, in turn, inhibits vascular smooth muscle relaxation and can be an effective method for refractory hypotension. Therefore, methylene blue can be used for the treatment of refractory vasoplegia unresponsive to other methods.

## Introduction

Methylene blue is commonly used for the treatment of methemoglobinemia and vasoplegia following coronary artery bypass procedures. The suggested mechanism of vasoplegia is a dysregulation of nitric oxide synthesis and vascular smooth cell guanylate cyclase activation [1]. Methylene blue is an inhibitor of guanylate cyclase, which decreases the production of cyclic guanosine monophosphate (cGMP). This, in turn, inhibits vascular smooth muscle relaxation and can be an effective method for the treatment of refractory hypotension [1]. Adverse effects of methylene blue include, most commonly, a blue-green discoloration of urine, as well as the possible development of serotonin syndrome when combined with serotonergic drugs and tricarboxylic acids. Methylene blue may also cause dizziness, confusion, headaches, or limb pain following IV administration. There are few contraindications to the use of methylene blue, including individuals with glucose-6-phosphate dehydrogenase (G6PD) deficiency, those who have demonstrated hypersensitivity or an anaphylactic reaction to administration, and those who are pregnant [2]. Although the only FDA-approved use of methylene blue is for methemoglobinemia, other off-label uses may be administered in necessary clinical scenarios. One such instance is a vasoplegic syndrome, which could have catastrophic outcomes if not acted upon promptly. 

Cardiovascular drugs have been implicated in a growing number of substance exposures by the American Poison Control Centers. Calcium channel blockers and beta blockers account for most deaths, and of the calcium channel blockers, verapamil was the most commonly reported [3]. The current treatment algorithm for patients who ingested a potentially toxic amount of calcium channel blockers but are asymptomatic recommends observation and consideration of decontamination with activated charcoal. For symptomatic patients, the first-line therapies include IV calcium, high-dose insulin therapy with other first-line therapies if evidence of myocardial dysfunction is present, norepinephrine, or epinephrine in the presence of shock [4]. Therapy for patients refractory to first-line treatments includes incremental doses of high-dose insulin or IV lipid-emulsion therapy [4].

## Case presentation

A 67-year-old female with a past medical history of hypertension, hyperlipidemia, congestive heart failure, atrial fibrillation, chronic obstructive pulmonary disease, obstructive sleep apnea, diabetes mellitus, seizures, anxiety, depression, and bipolar disorder presented to the ED with altered mental status following a suicide attempt. She was found down by her husband, surrounded by multiple pill bottles with varying amounts of remaining medications. The exact time of ingestion is unknown as the patient’s husband did not know how long she had been down, and the patient was unable to report the time of ingestion on presentation. The patient arrived with a bag of the medications she was found with, including metoprolol 50 mg, amlodipine 5 mg, mirtazapine 45 mg, pantoprazole 40 mg, quetiapine 50 mg, quetiapine 25 mg, levetiracetam 500 mg, melatonin 5 mg, levothyroxine 50 mg (Table 1). On arrival, the patient was hypotensive with blood pressure (BP) of 84/39 mmHg, heart rate of 84 beats per minute, respiratory rate of 12 breaths per minute, and oxygen saturation of 89%. She was placed on a non-rebreather with improvement in her oxygenation to 98%. She was protecting her airway and became alert with stimulation but was lethargic. The patient did report that she intentionally took medications approximately one hour prior to arrival but was not able to tell us what she took or how much she took. She received two liters of normal saline, but hypotension worsened with a BP of 56/20 mmHg. There was concern that the cause of her hypotension was due to an overdose of amlodipine. The patient did not appear to be bradycardic; QTc on EKG appeared normal, so there was a lower concern for metoprolol overdose. The patient received a total of three grams of calcium gluconate and was started on norepinephrine, followed by epinephrine. The patient required up to 70 mcg/min of norepinephrine and up to 20 mcg/min of epinephrine to meet a mean arterial pressure (MAP) goal above 65. She was transferred to the ICU, where she developed hypoxic and hypercarbic respiratory failure requiring intubation. Lab values obtained in the ED, which were relevant after transfer to the ICU, included the following: Cr 0.9 mg/dL, Cl 111 mmol/L, Na 141 mmol/L, K 3.6 mmol/L, CO_2_ 21 mmol/L, anion gap 8 mmol/L, glucose 117 mg/dL, AST 25 U/L, ALT 20 U/L, salicylate level <1 mg/dL, acetaminophen level <10 µg/mL, levetiracetam level 156.3 mcg/mL, pH 7.396, pCO_2_ 34.5 mmHg, O_2_ 70.3%, HCO_3_ 20.7 mmol/L, and EtOH <0.010 g/dL. She remained hypotensive on high doses of both norepinephrine and epinephrine. Vasopressin was added with ranges from 0.03 to 0.09 u/min followed by the addition of phenylephrine up to 100 mcg/min. The patient’s case was discussed with American Poison Control Centers, and per their recommendation, her ejection fraction was assessed by bedside echo. When this was determined to be normal, she was given a 1 mg/kg bolus of methylene blue. The patient was also started on D10, given a 1 unit/kg insulin bolus, and started on an insulin infusion at 0.5 units/kg/h. She was also started on stress-dose steroids. Nephrology was consulted on day two of admission due to steadily elevating creatinine to 1.18 mg/dL from the patient's baseline of 0.7-1.0 mg/dL. She had also developed a metabolic acidosis requiring a bicarbonate drip at 150 mL/h. At that time, continuous renal replacement therapy (CRRT) was deferred due to the patient’s hemodynamic instability, with concern that she would not tolerate any fluid removal. The patient’s case was discussed with a toxicologist, who recommended decreasing the insulin and dextrose infusion due to concerns that it might contribute to continued vasoplegia. She was given an additional methylene blue bolus of 2 mg/kg followed by a 1 mg/kg infusion. She was successfully weaned of phenylephrine on day three of her hospitalization. CRRT was initiated due to concern about fluid overload on day four of her stay and was continued for a three-day course to assist with volume removal. She was weaned off both epinephrine and vasopressin on day four; however, she required a short course of low-dose vasopressin over the next few days of her hospitalization (Table 2). Seven days after her initial presentation, the patient was successfully weaned off all vasopressor medications. The patient was subsequently extubated and evaluated by the psychiatry team. Fifteen days after her initial presentation, she was cleared for discharge to a long-term care facility in stable condition.

**Table 1:** Medications and dosages implicated as potential source of ingestion in polysubstance overdose.

Medication	Dose	Elimination half life
Amlodipine	5 mg	30-50 hours
Levetiracetam	500 mg	6-8 hours
Levothyroxine	50 mg	6-7 days
Melatonin	5 mg	20-40 minutes
Metoprolol	50 mg	3-7 hours
Mirtazapine	45 mg	20-40 hours
Pantoprazole	40 mg	1 hour
Quetiapine	25 mg	6-7 hours
Quetiapine	50 mg	6-7 hours

**Table 2:** Timeline of interventions given during hospitalization to maintain goal MAP > 65. MAP, mean arterial pressure; Q8H, every eight hours

Day of hospitalization	Medications	Lowest MAP recorded by arterial line per day	Fluid intake and output
Day 1	Norepinephrine 10-70 mcg/min, epinephrine 5-20 mcg/min, vasopressin 0.03-0.09 u/min, phenylephrine 50-100 mcg/min, methylene blue bolus 1 mg/kg, insulin bolus 1 u/kg, insulin infusion 0.5 u/kg/h, hydrocortisone sodium succinate 100 mg Q8H	35	I: 4,448.8, O: 700, I/O: +3,748.8
Day 2	Norepinephrine 10-80 mcg/min, epinephrine 5-20 mcg/min, vasopressin 0.04-0.09 u/min, phenylephrine 50-300 mcg/min, methylene blue bolus 2 mg/kg, methylene blue infusion 1 mg/kg/h, insulin infusion 0.5 u/kg/h, hydrocortisone sodium succinate 100 mg Q8H	55	I: 9,673, O: 1,220, I/O: +8,453
Day 3	Norepinephrine 25-40 mcg/min, epinephrine 5-17 mcg/min, vasopressin 0.02-0.06 u/min, phenylephrine 225 mcg/min-discontinued, hydrocortisone sodium succinate 100 mg Q8H	33	I: 4,493, O: 3,821, I/O: +672
Day 4	Norepinephrine 2-35 mcg/min, epinephrine 5 mcg/min-discontinued, hydrocortisone sodium succinate 100 mg Q8H	62	I: 1,891.7, O: 5,213, I/O: -3,321.3
Day 5	Norepinephrine 6-20 mcg/min, vasopressin 0.03 u/min, hydrocortisone sodium succinate 100 mg Q8H	62	I: 2,593.2, O: 7,491, I/O: -4,897.8
Day 6	Norepinephrine 8-20 mcg/min, vasopressin 0.03 u/min-discontinued, hydrocortisone sodium succinate 100 mg Q8H	63	I: 4,200.1, O: 6,250, I/O: -2,049.9
Day 7	Norepinephrine 10 mcg/min-discontinued, hydrocortisone sodium succinate 100 mg Q8H	52	I: 5,960, O: 5,250, I/O: +710

## Discussion

Calcium channel blockers are some of the most commonly used cardiovascular medications within the adult population and have a range of complications, some of which can be fatal. Calcium channel blockers work by targeting the L-type voltage-gated calcium channels, which are responsible for triggering myocardial contraction and maintaining vascular smooth muscle tone and insulin secretion [5]. Toxicity or overdose of these medications can cause significant bradycardia, hypotension, and conduction disturbances, reduce pancreatic insulin secretion, and induce end-organ insulin resistance [5]. Of this class of medications, amlodipine has a long half-life and strong vasodilatory effects, which are particularly concerning following an overdose [6].

Calcium channel blockers cause smooth muscle relaxation by the production of nitric oxide. L-arginine is transformed to nitric oxide by nitric oxide synthase. This then leads to the activation of soluble guanylyl cyclase and the production of cGMP. cGMP-dependent protein kinase has three main actions. It decreases the sensitivity of myosin to calcium-induced contractions, decreases calcium entry through calcium channels by activating calcium-sensitive potassium channels, and inhibits the release of calcium from the sarcoplasmic reticulum. These three factors combined contribute to the relaxation of smooth muscle [3]. Therefore, an overdose of these medications can lead to significant vasoplegia.

There are a variety of known ways to combat and treat calcium channel blocker overdose. Activated charcoal can be used to reduce gastrointestinal absorption when given within two hours after consumption [5]. Calcium administration may also be beneficial in promoting calcium influx through unblocked L-type calcium channels. In severe cases, catecholamine infusions can be used to combat refractory vasoplegia [5]. Methylene blue has been used as an off-label medication in these cases with some success.

Methylene blue decreases the amount of cGMP needed for the release of nitrous oxide, which causes vasoconstriction of blood vessels via inhibition of vascular smooth muscle relaxation [2]. Previous studies have shown methylene blue to increase pulse rate, MAP, and median survival time in amlodipine-induced shock in animal models [7]. Although there is no agreed-upon dose of methylene blue in the treatment of refractory vasoplegia, the best dosing regimen is suggested to be a 2 mg/kg bolus followed by a 0.25-2 mg/kg/h infusion [8]. This medication can be lifesaving, but it also has a number of adverse effects, some of which can be life-threatening. Side effects include localized skin necrosis at sites of prolonged peripheral intravenous administration, confusion, as well as headache, nausea, vomiting, abdominal pain, and at higher doses, hemolysis, severe intravascular hemolysis, hyperbilirubinemia, and death [9].

Contraindications for the use of this medication include pregnancy, as it may lead to fetal hypoxia, and G6PD deficiency, as it can precipitate hemolytic anemia [10]. There is also a US boxed warning for the use of methylene blue, as it may cause serious or fatal serotonergic syndrome when administered concomitantly with a selective serotonin reuptake inhibitor, selective serotonin reuptake inhibitor, monoamine oxidase inhibitor, or opioids. In the case of the patient discussed previously, there was an increased risk of developing serotonin syndrome with the use of methylene blue when combined with the implicated medications in the overdose. However, the decision was made to proceed with the medication, as the risk of possible serotonin syndrome was lesser than the risk of probable imminent death from untreated vasoplegia and severe hypotension. 

## Conclusions

Polysubstance overdose can make management of patients difficult when the culprit medication of the patient’s symptomology is unknown. When calcium channel blocker overdose is suspected as a potential cause of refractory vasoplegia, methylene blue should be considered as an adjunct treatment modality. Although, in this case, it is not possible to isolate methylene blue as the cause of her improved vasoplegia, some data suggest that it can work effectively as an adjunct in cases like this. Further research should be conducted to determine which treatment method is most useful in cases of refractory vasoplegia and compare the efficacy of methylene blue with other treatment options such as insulin and dextrose or more frequently used pressors: levophed, vasopressin, phenylephrine, epinephrine. Methylene blue can be used for the treatment of refractory vasoplegia when other methods of blood pressure support have been ineffective and should be considered as an adjunct in appropriate situations.
